# Incorporating terminal velocities into Lagrangian stochastic models of particle dispersal in the atmospheric boundary layer

**DOI:** 10.1038/s41598-018-34924-4

**Published:** 2018-11-15

**Authors:** Andy M. Reynolds

**Affiliations:** Rothamsted Research, Harpenden, Hertfordshire AL5 2JQ UK

## Abstract

Lagrangian stochastic models for simulation of tracer-particle trajectories in turbulent flows can be adapted for simulation of particle trajectories. This is conventionally done by replacing the zero-mean fall speed of a tracer-particle with the terminal speed of the particle. Such models have been used widely to predict spore and pollen dispersal. Here I show that this modification predicts that particles become uniformly distributed throughout the air column, which is at variance with the seminal experimental studies of Hirst *et al*. (1967) that demonstrated spore concentrations (and pollen concentrations) declined exponentially with height in unstable air. This discrepancy arises because the terminal speed, which is a Lagrangian property of a particle, has always been treated as if it were an Eulerian property of an ensemble of particles. In this study models are formulated correctly. I show that the mean acceleration of a tracer-particle should be replaced by the mean acceleration of a particle. Model predictions for aerial density profiles then agreed with the observations of Hirst *et al*. (1967) and with observations of ground-level concentrations but differed significantly from predictions obtained using conventional models. In accordance with the results of numerical simulations, the models also predict that particles are moving downwind marginally more slowly than the wind itself. Finally, the new modelling approach can be extended to predict the dispersal of small insects with active flight behaviours.

## Introduction

In his seminal work, Thomson^1^ correctly noted that turbulent dispersal within complex, inhomogeneous turbulent flows, such as the atmospheric boundary-layer, is best predicted by Lagrangian stochastic models because other methods (e.g., diffusion equations and similarity theory) are either invalid or inappropriate. Thomson^1^ showed how Lagrangian stochastic models for simulation of tracer-particle trajectories could be formulated by invoking the ‘well-mixed condition’. Thomson’s ‘well-mixed condition’ states that: If, at time *t* = *t*_0_, the joint distribution of tracer-particle positions (**x**) and velocities (**u**), *P*(***u***, ***x***, *t*), is proportional to the Eulerian joint distribution of positions and velocities, *P*_*E*_ (*u*, *x*, *t*), then at all later times, (*t* > *t*_0_), *P*(***u***, ***x***, *t*) must remain proportional to *P*_*E*_ (***u***, ***x***, *t*). This condition (assumption) is equivalent to, or more stringent than, all other criteria that, to date, have been identified as being capable of distinguishing between well- and poorly-formulated models. Mathematically, it requires that the model is derived from a Fokker-Planck equation. Lagrangian stochastic models satisfying the ‘well-mixed condition’ do accurately predict tracer-particle dispersal in: plant canopy turbulence; fully-convective boundary-layers; and other complex, inhomogeneous turbulent flows^2–5^. Nonetheless, there is currently no rigorous theoretical framework for formulation of Lagrangian stochastic models that is equivalent to the ‘well-mixed condition’ and capable of simulating heavy particle trajectories^6^. In the absence of such a framework, *ad hoc* modifications have been made to tracer-particle models to account for the effects of falling under gravity and particle inertia. Here I show that the most used, and seemingly most intuitive of these modifications, is inconsistent with the classic observations of Hirst *et al*.^7^ for pollen dispersal in the atmospheric boundary-layers. I then formulate new models that overcome this problem for small particles such as spores and pollen grains. This is of practical significance because model predictions for ground-level concentrations and dispersal ranges differ significantly from predictions obtained using conventional models.

## Methodology and Results

### Preliminary illustrative analysis: Particles in homogeneous turbulence

For illustrative purposes, I began with a one-dimensional analysis of the simplest case of a small, rigid, much-denser-than-air, spherical particle falling through isotropic homogeneous turbulence with Gaussian velocity statistics. The dominant forces on particles of this type are Stokes drag and gravity. The one-dimensionality of the analysis mirrors the modelling of tracer-particle trajectories in boundary layers where Lagrangian stochastic models are frequently used to model vertical movements due to turbulence, and where turbulence is assumed to have a negligible effect on downwind movements which are dominated by the mean flow (advection). In common with the formulation of Lagrangian stochastic models for simulation of tracer-particle trajectories^1^ I have assumed that the turbulent velocities experienced by particles along their trajectories can be represented as continuous Markov processes. For Lagrangian stochastic models this is appropriate within the large Reynolds number limit. This is because the Lagrangian acceleration autocorrelation function approaches a δ function at the origin, corresponding to an uncorrelated (white noise) component in the acceleration, and hence to a Markov process^8^. The white noise does, however, result in an exponential autocorrelation function that is non-differentiable at *t* = 0. This non-analytic behaviour can be alleviated by introducing a second timescale into the model, the Kolmogorov time scale, *t*_*η*_, i.e., by replacing the white noise with coloured noise, that is exponentially correlated on a time scale, *t*_*η*_ (in this case the first and second but not higher derivatives of the autocorrelation function are defined at *t* = 0)^8^. I did not attempt this here because its impact on long-time dispersion statistics is expected to be negligible at high Reynolds numbers^8^, and because the accurate representation of the smallest scales of turbulence experienced by particles is problematic^9^. The tendency for preferential congregation of particles along ‘fast tracks’ at the boundaries of vortices results in an increase in the average settling velocity^10,11^, which is also neglected. Preferential sampling of turbulent flows is not significant when Stokes numbers, *S*_*t*_ = *τ*/*t*_*η*_, are much less than one^11^, as is the case here where the focus is on spores and pollen that are dispersing in atmospheric boundary-layers. The aerodynamic response times of spores and pollen grains, *τ*, is about 10^−4^ s and so shorter than *t*_*η*_ which is about 10^−1^ s.

With the aforementioned assumptions, the governing equation for motion in a vertical direction can be obtained by coupling Stokes’ law and gravitational settling with an Ornstein–Uhlenbeck process that models the turbulent flows experienced by the particle:

1 $$\begin{array}{rcl}\frac{dv}{dt} & = & {\tau }^{-1}\,(u-v)+g\\ \frac{du}{dt} & = & -\,{T}^{-1}\,u+\sqrt{2{\sigma }_{u}^{2}{T}^{-1}}\dot{W}\\ \frac{dx}{dt} & = & v\end{array}$$

where *x* and *v* are the position and velocity of the particle at time *t*; *u* is the fluid velocity in the immediate vicinity of the particle; *g* is acceleration due to gravity; and $\dot{W}$ denotes a white noise process, which has the property ${\int }_{0}^{t}\dot{W}(t^{\prime} )\,dt^{\prime} =W(t)$ for which *W*(*t*) is a Wiener process. The modelled velocities, u, are Gaussian distributed with mean zero and variance ${\sigma }_{u}^{2}$, and they are exponentially-distributed on a timescale *T* that is representative of the energy-containing scales of motion. It is then apparent that the average settled velocity (a Lagrangian velocity evaluated at the particle location) $\langle v\rangle =\tau g$. Here the model is used with reflective boundary conditions at ground level to create an impenetrable barrier to transport. These conditions ensure maintenance of equilibrium position- and velocity-statistics, thereby allowing any analytical predictions developed below to be tested^12^. Note also that the dispersiveness of the smallest spores (with diameters <10 µm) is so high that deposition occurs very infrequently^13^.

Below I describe how the modelling approach is refined to account for inhomogeneous turbulent velocity statistics and for multi-dimensional turbulence which are both important near ground level^3^.

**Figure 1:**
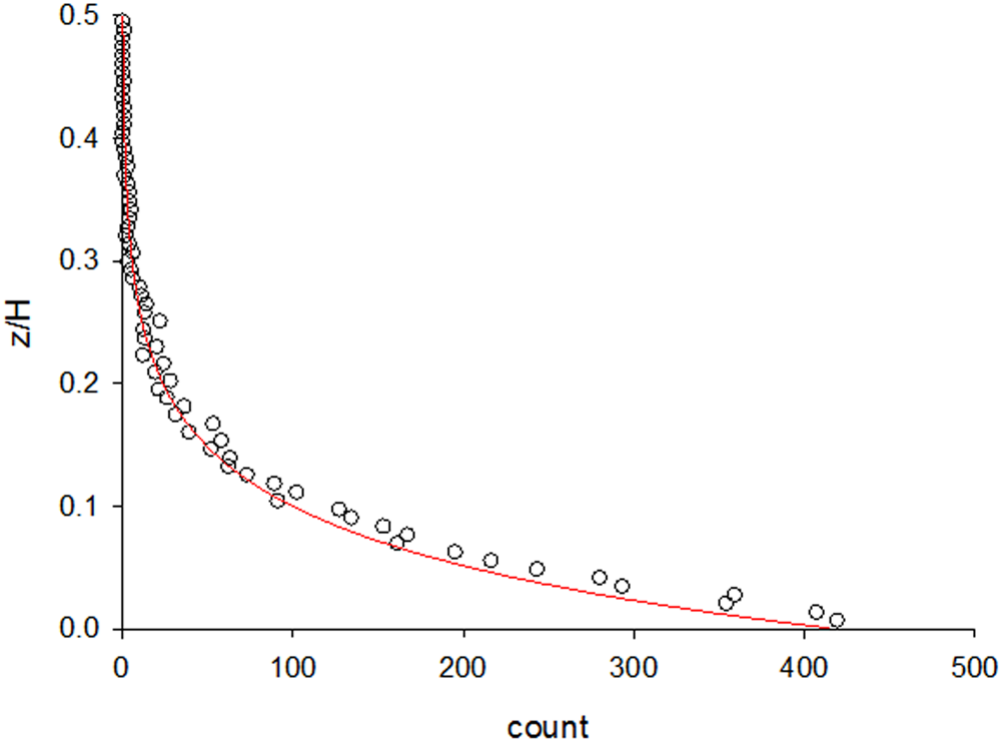
Predicted aerial density profiles of particles in 1-dimensional homogeneous turbulence (●) together with the theoretical prediction (red line) of Eq. 6. The trajectories of 5000 particles were simulated using Eq. 1 with, *τ* = *1*, *T* = *10*, ${\sigma }_{u}^{2}=1$ and *g* = *−0.1 a*.*u*. Reflective boundary-conditions were applied at *x*_*1*_ = *0* and *x*_*1*_ = *H* = *1000*. Particles were released from *x*_*1*_ = *50*. Each particle was simulated for a time *72*,*000 a*.*u*. after which its position was recorded.

The model predicts that equilibrium numbers of aerosol particles will decrease exponentially with height (Fig. 1) in accordance with the observations of Hirst *et al*.^7^. This decrease is a consequence of the imposition of an impenetrable boundary at ground level and would not arise in open systems that do not have such boundary conditions. In open systems, particles become uniformly distributed in the presence of a sustaining influx of particles, or in the presence of periodic boundary conditions. When the equilibrium distribution of particles decreases with height there is a balance between a relatively large number of particles moving upwards, which are slowed by gravity, and a relatively small number of particles moving downwards and being accelerated by gravity. In other words, the Eulerian velocity of many particles is zero at a given location. Nonetheless, the average Lagrangian velocity of any particular particle is its’ terminal speed, *τg*. This distinction between Eulerian and Lagrangian velocities does not arise in open systems but, as shown below, is of crucial importance in the formulation of Lagrangian stochastic models for simulation of heavy particle trajectories (which use Eulerian velocity statistics as model inputs).

Stokes’ law and the Ornstein–Uhlenbeck process (Eq. 1) can be combined into a single equation:

2 $$\begin{array}{rcl}\frac{{d}^{2}v}{d{t}^{2}} & = & {\tau }^{-1}(\frac{du}{dt}-\frac{dv}{dt})\\  & = & -{\tau }^{-1}\,\frac{dv}{dt}-{\tau }^{-1}{T}^{-1}u+\sqrt{2{\sigma }_{u}^{2}{T}^{-1}{\tau }^{-2}}\dot{W}\\  & = & -{\tau }^{-1}\,\frac{dv}{dt}-{\tau }^{-1}{T}^{-1}(\tau \frac{dv}{dt}-\tau g+v)+\sqrt{2{\sigma }_{u}^{2}{T}^{-1}{\tau }^{-2}}\dot{W}\\  & \equiv  & -({\tau }^{-1}+{T}^{-1})(\frac{dv}{dt}-\frac{\tau }{T+\tau }g)-{\tau }^{-1}{T}^{-1}v+\sqrt{2{\sigma }_{v}^{2}({T}^{-1}+{\tau }^{-1}){T}^{-1}{\tau }^{-1}}\dot{W}\\  & \equiv  & a(A=\frac{dv}{dt},v,x)+b\dot{W}\end{array}$$

where ${\sigma }_{v}^{2}=\frac{{\sigma }_{u}^{2}}{1+\tau /T}$. This shows that Eq. 1 is effectively a second-order autoregressive stochastic process^8^ in which the position, velocity and acceleration of the particle are modelled collectively as a Markovian process. The equilibrium Eulerian distribution of position, velocity and acceleration, *P*(*A*, *v*, *x*), can be obtained from the associated Fokker-Planck equation^10^:

3 $$\frac{\partial P}{\partial t}+v\frac{\partial P}{\partial x}+A\frac{\partial P}{\partial v}=-\,\frac{\partial }{\partial A}(aP)+\frac{{b}^{2}}{2}\frac{{\partial }^{2}P}{\partial {A}^{2}}$$

The Fokker-Planck equation (Eq. 3), is the Eulerian counter-part of the Lagrangian model (Eq. 1)^14^. It follows from Eq. 3 that Eulerian velocities (evaluated at a fixed location) are Gaussian distributed with mean zero and variance ${\sigma }_{v}^{2}$ and that Eulerian accelerations are Gaussian distributed with mean $\langle A\rangle =\frac{\tau g}{\tau +T}\approx \frac{\tau }{T}g$ and variance ${\sigma }_{A}^{2}=\frac{{\sigma }_{v}^{2}}{\tau T}$ so that

4 $$P(A,v,x)=\frac{\rho (x)}{2\pi {\sigma }_{v}{\sigma }_{A}}\exp (-\frac{{v}^{2}}{2{\sigma }_{v}^{2}})\exp (-\frac{{(A-\langle A\rangle )}^{2}}{2{\sigma }_{A}^{2}})$$

An equation for $\rho (x)$ can be obtained from Eq. 3 by integrating over all accelerations:

5 $$\frac{\partial p}{\partial t}+v\frac{\partial p}{\partial x}+\langle A\rangle \frac{\partial p}{\partial v}=0$$

where $p(x,v)=\frac{\rho (x)}{\sqrt{2\pi }{\sigma }_{v}}\exp (-\frac{{v}^{2}}{2{\sigma }_{v}^{2}})$. It follows that Eq. 5 has the stationary solution:

6 $$\rho (x)={\rho }_{0}\,\exp (\frac{x\tau g}{{\sigma }_{v}^{2}T}).$$

This prediction is consistent with Hirst *et al*.^7^ who reported that spore (and pollen) concentrations often declined exponentially with height. It is further supported by the results of numerical simulations, obtained using Eq. 1 (Fig. 1). As mentioned above, the exponential aerial density profile (Eq. 6) reconciles the apparent contradiction between zero mean Eulerian velocities and non-zero mean Eulerian accelerations. This is at variance with standard models of particle dispersal^6,15^ which corresponds to a seemingly trivial re-arrangement of the terms in the penultimate line of Eq. 2:

$$\frac{{d}^{2}v}{d{t}^{2}}=-\,({\tau }^{-1}+{T}^{-1})\frac{dv}{dt}-{\tau }^{-1}{T}^{-1}(v-\tau g)+\sqrt{2{\sigma }_{v}^{2}({T}^{-1}+{\tau }^{-1}){T}^{-1}{\tau }^{-1}}\dot{W}$$

but which effectively assumes that the average Eulerian velocity $\langle v\rangle =\tau g$, and that the Eulerian acceleration $\langle A\rangle =0$. It can be seen by integrating Eq. 5 over all velocities that these conventional assumptions correspond to uniform equilibrium density profiles, $\rho (x)={\rho }_{0}$, that are incompatible with the observations of Hirst *et al*.^7^, but are consistent with expectations of open systems.

These different model predictions are of considerable practical importance because they lead to significant differences in predicted ground-level concentrations and dispersal ranges. Note, for example, that the assumption that air density within deep planetary boundary layers is constant is incorrect. In fact, the density at the top of the boundary-layer top may be more than 20% lower than at the surface, which leads to errors in the order of 10% in the tracer concentrations^16^. In the presence of a vertical wind shear, this also leads to inaccurate calculations of horizontal tracer transport. Much larger errors are expected for particles; Hirst *et al*.^7^ reported 100-fold changes in spore and pollen concentrations.

The prediction made in Eq. 6 is consistent with the seminal observations of Hirst *et al*.^7^ which reported that, in unstable air, spore (and pollen) concentrations generally declined exponentially with height but were sometimes almost uniform in the lowest kilometre where mixing was strong (i.e., where the diffusivity, ${\sigma }_{v}^{2}T$, was large). Analogous results were recently reported by Zhang *et al*.^17^. Moreover, observed exponential decay rates^7^ are consistent with the theoretical expectations of spores in atmospheric turbulence.

### Particles in heterogeneous turbulence

A first-order autoregressive stochastic process in which the position and velocity of a particle are modelled collectively as a Markovian process, can be obtained by multiplying both sides of Eq. 2 by *τ* and then taking the limit $\tau /T\to 0$, with *T* and the settling velocity, $\tau g$, both fixed:

7 $$\frac{dv}{dt}=-\,v{T}^{-1}+\tau {T}^{-1}g+\sqrt{2{\sigma }_{u}^{2}{T}^{-1}}\dot{W}$$

which corresponds to the Eulerian distribution:

8 $$P(v,x)=\frac{{\rho }_{0}}{\sqrt{2\pi }{\sigma }_{u}}\exp (-\frac{{v}^{2}}{2{\sigma }_{u}^{2}})\exp (\frac{x\tau g}{{\sigma }_{u}^{2}T}).$$

This shows that, for homogeneous Gaussian turbulence, the model that is appropriate for simulation of tracer-particle trajectories (i.e. the Ornstein–Uhlenbeck process) can also be an effective model for simulation of small-particle trajectories when an acceleration term, $\tau {T}^{-1}g$, is added. This suggests that models for simulation of tracer-particles in heterogeneous turbulence could also be modified in a directly analogous way:

9 $$\frac{dv}{dt}=-\,v{T}^{-1}+\frac{1}{2}[1+\frac{{v}^{2}}{{\sigma }_{u}^{2}}]\frac{d{\sigma }_{u}^{2}}{dx}+\tau {T}^{-1}g+\sqrt{2{\sigma }_{u}^{2}{T}^{-1}}\dot{W}$$

I make no attempt here to model turbophoresis (the tendency of particles to concentrate preferentially in regions of flow with relatively low turbulent kinetic energy) because this is a weak tendency when *τ*/*T* → 0. Moreover, I assume that ${\sigma }_{v}^{2}\approx {\sigma }_{u}^{2}$ is appropriate when *τ*/*T* → 0, which is the case here. The model (Eq. 9) is identical to the one proposed by Stohl and Thomson^15^ for tracer-particle trajectories in deep planetary boundary-layers; Eq. 9 corresponds to the Eulerian distribution:

10 $$P(v,x)=\frac{{\rho }_{0}}{\sqrt{2\pi }{\sigma }_{u}}\exp (-\frac{{v}^{2}}{2{\sigma }_{u}^{2}})\exp (\tau g{\int }_{0}^{x}\frac{dx}{{\sigma }_{u}^{2}T})$$

**Figure 2:**
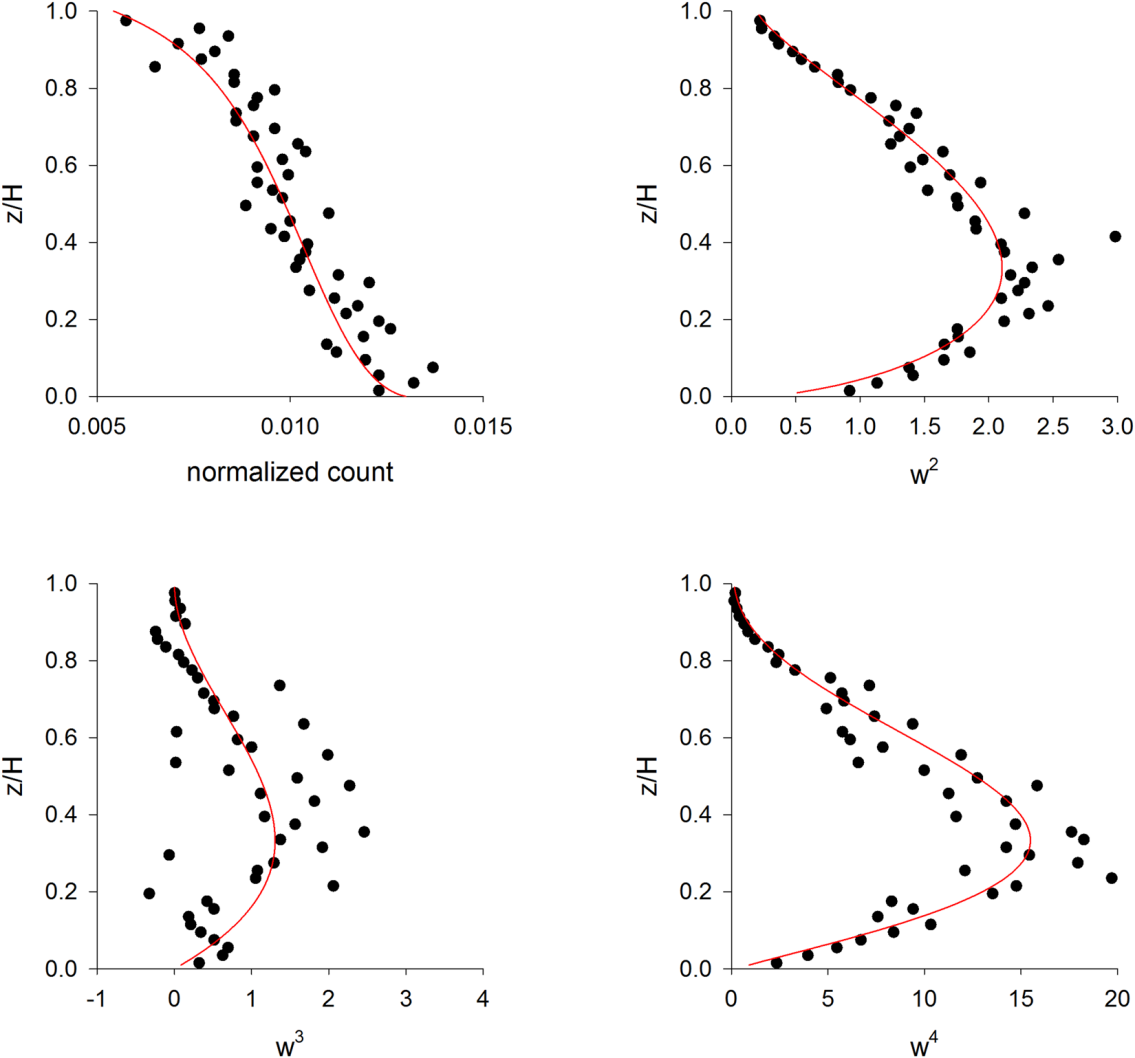
Predicted aerial density profile and Eulerian velocity statistics of particles in a purely convective boundary-layer (●) are consistent with model inputs (red lines). The trajectories of 10,000 particles were simulated using the Lagrangian stochastic model of Franzese *et al*.^4^ with an additional term representing acceleration due to gravity, $\tau g/T$. Each particle was simulated for *10*,*000* *s* after which its position and velocity were recorded. Predictions were obtained for a boundary-layer with height H = 1000 m and convective velocity scale w* = *2*.*0* *ms*^−*1*^, and for particles *τg*/*T* = *0*.*001* *ms*^−*2*^. This agreement is specific to the model of Franzese *et al*.^4^ as comparable predictions (not shown) were obtained using the Lagrangian stochastic model of Luhar *et al*.^2^ which has a more complicated mathematical structure.

As such, Eq. 9 predicts a marked decline at the height of the inversion when turbulent diffusivity ${\sigma }_{u}^{2}T$ becomes weak, in accordance with the observations of Hirst *et al*.^7^. This suggests that Lagrangian stochastic models for simulation of tracer-particle trajectories in non-Gaussian turbulence (i.e., for convective flows and plant canopy turbulence^2,18^) could be modified in a similar way. The addition of a gravity term is not entirely self-consistent if the underlying stochastic model is founded on non-Gaussian velocity statistics, because simulated velocities (model outputs) would no longer be compatible with model inputs. Nonetheless, results from numerical simulations of spore movements in a convective boundary-layer reveal that the modification, although not exactly self-consistent, does not result in significant differences between Eulerian distributions of velocity (which are used as model inputs) and predicted (outputted) Eulerian distributions of velocity (Fig. 2). Precise consistency requires that the gravity term be speed dependent, i.e., it requires that particles actively respond to turbulence which is incompatible with the notion of a passive particle. Breakdown of the approximation for strong non-Gaussian turbulence might simply reflect the fact that model outputs (particle velocities) can no longer be accurately characterised in terms of fluid-velocity statistics (which are used as model inputs). It should also be noted that no reflection scheme is exactly consistent with the modelling when applied at locations where the pdf for the normal velocity is asymmetric or is locally-inhomogeneous^19^. This, however, does not prohibit the existence of reflection algorithms that are acceptable in practice. Moreover, because the statistical character of the flow close to the boundary is unknown, profiles of flow statistics at the boundary can be selected to ensure successful application of the reflection algorithm.

### Accounting for turbulence in a stream-wise direction

The analysis described previously can be extended to account for cross correlation between turbulent fluctuations in stream-wise and vertical directions, which is important near to the ground^3^. In this case, Eq. 1 is replaced by:

11 $$\begin{array}{c}\frac{d{v}_{i}}{dt}={\tau }^{-1}({u}_{i}-{v}_{i})+g{\delta }_{i2}\\ \frac{d{u}_{i}}{dt}=-\,{\frac{b}{2}}^{2}{\lambda }_{ij}u+b{\dot{W}}_{i}\\ \frac{d{x}_{i}}{dt}={v}_{i}\end{array}$$

where the subscripts denote Cartesian coordinates. *δ*_*ij*_ is a Kronecker delta indicating that gravity is acting in the 2-direction and *λ*_*ij*_ is an element of the inverse of the velocity covariance matrix. In Lagrangian stochastic modelling the parameter *b* is usually selected so that model predictions for the Lagrangian velocity structure are consistent with Kolmogorov’s similarity theory^1^. This requires that $b=\sqrt{{C}_{0}\varepsilon }$ where *C*_0_ is a universal constant and *ε* is the mean rate of dissipation of turbulent kinetic energy. Repeating the steps above leads to generalization of Eq. 7 to form:

12 $$\frac{d{v}_{i}}{dt}=-\,\frac{{b}^{2}}{2}{\lambda }_{ij}{v}_{j}+\frac{{b}^{2}}{2}{\lambda }_{i2}\tau g+b{\dot{W}}_{i}$$

which can be written more explicitly as:

13 $$\begin{array}{rcl}\frac{d{v}_{1}}{dt} & = & -\frac{{b}^{2}}{2}{\lambda }_{11}({v}_{1}-\frac{{\lambda }_{12}}{{\lambda }_{11}}\tau g)-\frac{{b}^{2}}{2}{\lambda }_{12}{v}_{2}+b{\dot{W}}_{1}\\ \frac{d{v}_{2}}{dt} & = & -\frac{{b}^{2}}{2}{\lambda }_{12}({v}_{1}-\frac{{\lambda }_{12}}{{\lambda }_{11}}\tau g)-\frac{{b}^{2}}{2}{\lambda }_{22}{v}_{2}+\frac{{b}^{2}}{2}\tau g({\lambda }_{22}-\frac{{\lambda }_{12}^{2}}{{\lambda }_{11}})+b{\dot{W}}_{2}\end{array}$$

**Figure 3:**
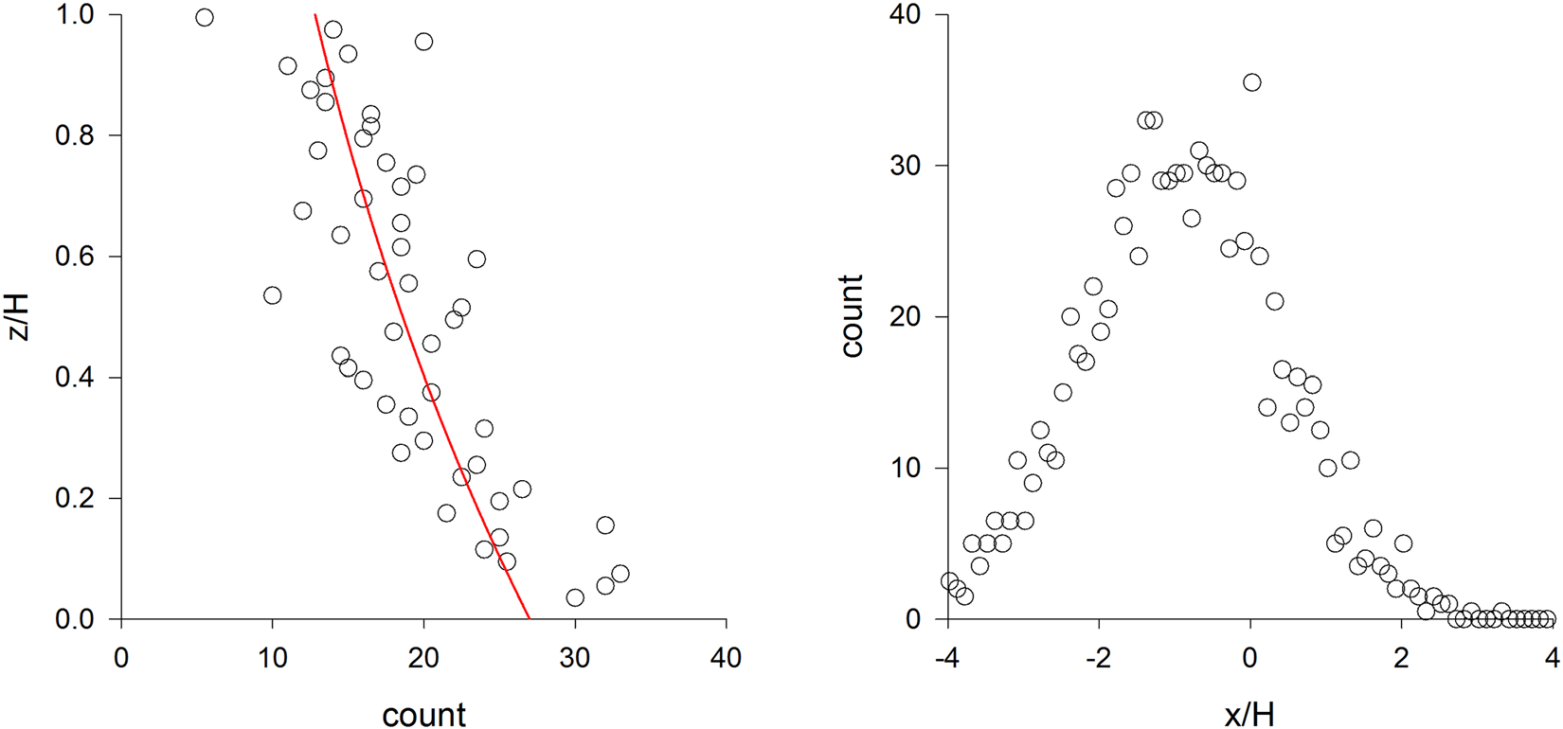
Predicted aerial density profiles of particles in 2-dimensional homogeneous turbulence (●) together with the theoretical prediction (red line). The trajectories of 2000 particles were simulated using Eq. 11 with ${b}^{2}=0.02,\,\langle {u}_{1}{u}_{1}\rangle =\langle {u}_{2}{u}_{2}\rangle =1.0$, $\langle {u}_{1}{u}_{2}\rangle =-\,0.5,\,\tau =1.0$ and *g* = *−0*.*1 a*.*u*. Reflective boundary-conditions were applied at *x*_*1*_ = *0* and *x*_*1*_ = *H* = *1000*. Particles were released from (*x*_*1*_, *x*_*2*_) = (*0*, *50*). Each particle was simulated for a time *10*,*000 a*.*u*. after which its position was recorded. Notice that the particles have been displaced backwards from their initial positions. The mean horizontal velocity of the simulated particle −0.07 is close to the theoretical expectation $\langle {v}_{1}\rangle =\frac{{\lambda }_{12}}{{\lambda }_{11}}\tau g=-\,0.05$ from Eq. 13.

It is evident that the mean velocity has components $\langle {v}_{1}\rangle =\frac{{\lambda }_{12}}{{\lambda }_{11}}\tau g$ and $\langle {v}_{2}\rangle =0$, and that the components for mean accelerations are $\langle {A}_{1}\rangle =0$ and $\langle {A}_{2}\rangle =\frac{{b}^{2}}{2}\tau g({\lambda }_{22}-\frac{{\lambda }_{12}^{2}}{{\lambda }_{11}})$. It follows from analysis of the corresponding Fokker Planck equation (see above for methodology) that $\rho ({x}_{2})={\rho }_{0}\,\exp (\frac{{b}^{2}}{2}\tau g\frac{{\lambda }_{22}}{\langle {u}_{2}{u}_{2}\rangle }{x}_{2})$ and that particles eventually become uniformly distributed in the horizontal (x_1_) direction. Just as in the 1-dimensional case, at any given location there is a balance between a relatively large number of particles moving upwards and being slowed by gravity, and a relatively small number of particles moving downwards and being accelerated by gravity. However, because the correlation, *λ*_12_, changes in the vertical, components of velocity are accompanied by changes in the horizontal components of velocity resulting in a non-zero mean horizontal velocity, 〈v_1_〉. Of course, components of the term $\frac{{b}^{2}}{2}{\lambda }_{i2}\tau g$ can be distributed differently, e.g., to give $\langle {A}_{i}\rangle =\frac{{b}^{2}}{2}{\lambda }_{i2}\tau g$. However, these redistributions correspond to exponential distributions of particle concentrations in the stream-wise direction, which could only be realized if there was a non-physical reflective boundary-condition at *x*_1_ = 0. The only other way to address this, which is also the most intuitive, is to use $\langle {v}_{1}\rangle =0,\,\langle {v}_{2}\rangle =\tau g,\,\langle {A}_{1}\rangle =\langle {A}_{2}\rangle =0$, which corresponds to a uniform or decaying aerial density profile (see analysis of 1-dimensinal model for explanation). The results of numerical simulations using Eq. 11 (Fig. 3) confirmed the key prediction of Eq. 13 which is that particles tend to accelerate downwards but do not increase their speed (this is not a contradiction, see analysis of the one-dimensional model for explanation) and that, as a result of the coupling between gravity and turbulence, the average downwind particle velocity is less than the downwind velocity of the surrounding air flow. For spores in the atmospheric boundary-layer this difference is predicted to be about 0.01 ms^−1^. This tendency should not be confused with that reported by Kaftori *et al*.^20^ for particles in near-wall turbulence. Kaftori *et al*.^20^ observed that these particles were often concentrated in regions of low velocity and associated with wall structures; as a result the average particle velocity was lower than the surrounding air. Coherent flow structures do not feature in the current theoretical analysis.

## Discussion

There is no rigorously correct theoretical framework for formulation of models that simulate particle trajectories in turbulent flows. The prevailing view is that particles fall through turbulent airstreams under gravity and that the force of gravity is eventually balanced by drag whereupon the particles stop accelerating and begin falling at their terminal velocity. Consequently, models that simulate tracer-particle trajectories have been adapted to simulate particle trajectories by simply neglecting particle inertia and by superimposing the particle’s settling velocity onto the fluid velocity^6,14^. In this study I have shown that these models are at variance with the seminal studies of Hirst *et al*.^7^ and with subsequent studies^17^ showing that aerial density profiles generally decrease exponentially with height. This discrepancy between predictions and observations has arisen because the terminal velocity, a Lagrangian property of particles, has been treated as if it were an Eulerian property of an ensemble of particles. I have shown that the mean acceleration of a tracer-particle should, in fact, be replaced by the mean acceleration of a particle. This suggests that conventional models are flawed fundamentally and can be expected to under-predict ground-level concentrations and over-predict dispersal; this is because simulated particles are uniformly distributed throughout the boundary-layer rather than preferentially concentrated close to the ground. The addition of an acceleration term (i.e., gravity) to Lagrangian stochastic (particle trajectory) models was entirely self-consistent when turbulence was Gaussian (i.e., when the stability is ideally neutral) and works well when turbulence was non-Gaussian (i.e., when the stability is fully convective).

The observations of Hirst *et al*.^7^ mirror those of Johnson^21^ and his collaborators which were made in the late 1940s and 1950s; they observed that aerial concentrations of aphids and other small insects decreased with height according to a power-law, rather than in an exponential way as reported for spores and pollen. In the case of Gaussian turbulence, dispersal of small insects can be modelled in an analogous way by adding a term to Lagrangian stochastic models that simulates height-dependent gravity (and which would not be out-of-place for a passive particle in ‘flatland’). For non-Gaussian turbulence (i.e., for convective conditions) the simulated gravity must also be speed-dependent but its effect on dispersion can be accurately approximated using a speed-averaged form^22^. Averaging this term over all velocities resulted in a gravity-like term, albeit a height-dependent one (which for homogeneous turbulence decreased with height by 1/x). The results of numerical simulations (not shown) revealed that the height-dependent gravity term was a useful approximation that did not typically result in significant differences between Eulerian distributions of velocity (which are used as model inputs) and predicted (outputted) Eulerian distributions of velocity. This realization potentially unifies the modelling of spores and the modelling of pests.
